# Does Repeating CT-Guided Transthoracic Fine Needle Aspiration Increase Diagnostic Yield and Complication Rate? A Single Institution Experience

**DOI:** 10.5812/iranjradiol.10031

**Published:** 2013-05-20

**Authors:** Esra Yazar, Funda Seçik, Pınar Yıldız

**Affiliations:** 1 Pulmonary Medicine Department, Yedikule Chest Disease Education and Training Hospital, Yedikule, İstanbul, Turkey

**Keywords:** Fine Needle Aspiration Biopsy, Invasive Procedure, Pneumothorax

## Abstract

**Background:**

Transthoracic fine needle aspiration biopsy is a well-established and safe technique for obtaining pulmonary tissue. However, there is very little data about repeating procedure.

**Objectives:**

We aimed to investigate whether repeating CT-guided transthoracic fine needle aspiration (TFNA) increases diagnostic yield and complication rate.

**Patients and Methods:**

Patients underwent TFNA and the final diagnoses achieved were included in the study. Consequently, 316 TFNA procedures performed in 240 patients were investigated retrospectively. A diagnosis was not reached in the first TFNA in 64 patients, then they underwent repeated TFNA. The factors that affected the diagnostic yield and complication rate were recorded.

**Results:**

The final diagnoses of 199 (82.9%) patients were malignant and 41 patients were benign. One hundred seventy six patients underwent the TFNA procedure only once. Sixty-four patients underwent a second procedure, while 12 underwent a third one. The diagnosis rate in the first procedures (diagnosis obtained in 142 out of 240 patients) was 59%. With the repeated procedures, 30 other patients were diagnosed. The diagnosis rate increased to 72% (172 out of 240 patients) (P<0.001). Twenty-nine (9.2%) pneumothoraces in 26 patients were detected in 316 TFNA procedures. In the repeated TFNA group (64 patients) there were seven pneumothoraces (11%) in the first TFNA procedure and six pneumothoraces (9%) in the repeated TFNA procedures (P=0.41). In three patients, pneumothorax was detected in the first and repeated procedures. Pneumothorax was significantly associated with the maximum diameter of the lesion (P=0.003), distance to pleura (P=0.001), contact to the pleura (P=0.0001) and smoking history (pack/year) (P=0.04).

**Conclusion:**

This study demonstrated that repeating the TFNA procedure in pulmonary lesions improves the diagnostic yield without an increase in the rate of pneumothorax.

## 1. Background

Transthoracic fine needle aspiration (TFNA) biopsy is a well-established and safe technique to obtain pulmonary tissue for pathologic examination. The diagnostic rate of TFNA ranges from 76% to 97% for malignant lesions (1-3). However, it has a low diagnostic rate for benign lesions that varies from 12% to 68% (4). Pneumothorax is the most frequent complication of TFNA biopsy. Reported rates range from 7.6% to 46% (2, 5). Parenchymal hemorrhage and hemoptysis occur less frequently. Systemic air embolism or pericardial tamponade are rare, but very serious complications (4, 6).

## 2. Objectives

Up to now, studies have investigated many factors associated with the diagnostic rate and complications of TFNA. In this study, we investigated the influence of repeating TFNA on diagnostic yield and complication rate.

## 3. Patients and Methods

Three hundred sixteen consecutive TFNA were performed on 240 patients in our institution, between January 2007 and June 2010 and were retrospectively evaluated. A diagnosis was not reached in the first TFNA in 64 patients, who then underwent a second procedure, while 12 underwent a third one. A detailed clinical history, demographic data, history of tuberculosis contact and smoking habit was obtained. The factors which could affect the diagnostic yield and complication rates were recorded. Informed consent was obtained from all patients prior to the procedure. For a solitary nodule or mass, the indication for TFNA depended upon the pretest probability of malignancy, and the presence or absence of metastatic disease. Another indication for TFNA included evaluation of a mediastinal mass after failed or negative broncoscopy. TFNA was performed for the evaluation of infectious processes presenting as nodules or mass especially in smokers. Patients with severe chronic obstructive pulmonary disease (FEV1 < 1L), previous pneumonectomy, arteriovenous malformation, aneurysm, pulmonary hypertension and abnormal coagulation tests did not undergo TFNA procedure. The patients underwent TFNA and the final diagnosis was included in the study. The biopsies were performed by a pulmonologist who had at least a five-year experience on this procedure. The patients were placed in prone or supine positions, depending on the location of the lesion. At the time of biopsy, CT images were obtained at a 5 mm section thickness throughout the lesion. Localization was determined by CT images with laser lighting and skin markers by a radiology technician and the pulmonologist. The mean depth of the lesions was measured from the pleural surface to the most distal tumor limit. All biopsies were performed by a 22-gauge 9 or 15-cm-long Chiba needle. A 50 mL syringe was used to aspirate the material. Cytopathology samples (slides and cell blocks) were prepared by a pathology technician at the time of aspiration. If tuberculosis was suspected, the smears were stained using the Ziehl-Neelsen method and culture for Mycobacterium tuberculosis was performed. Following the biopsy, immediate CT was obtained to check for pneumothorax. Chest x-ray was obtained 2-4 hours after the procedure or earlier if the patient became symptomatic. Cases developing pneumothorax were followed up in the hospital. Statistical analysis was performed by SPSS 11.5 for Windows (SPSS Inc., Chicago, Ill, USA). Categorized data were analyzed by Pearson Chi Square and Fisher's Exact tests and numeric data were analyzed by Student's t test. Binominal test was used to compare the pneumothorax and diagnosis rate in the repeated attempts. P value less than 0.05 was considered significant.

## 4. Results

Three hundred sixteen consecutive TFNAs were performed in 240 patients. The study population included 216 (90%) males and 24 females (10%), with a mean age of 59±12 years (range, 17- 81 years). The amount of smoking was 47±29 pack/year. One hundred ninety two patients were current smokers, 35 patients were ex-smokers and 13 patients had never smoked. General features of the procedures are given in Table 1.

**Table 1.** General Features of Procedures and Lesions

Features of Procedures and Lesions	Procedures (n=316)
Needle İnsertion Angle: Perpendicular/Oblique, No. (%) (n=316)	252 (80)/64 (20)
Position: Prone/Supine, No. (%) (n=316)	179 (57)/137 (43)
Lesion Size, mean ± 2 SD, mm (range)	50.5 ± 22.6 (10-120)
Lesion Depth, mean ± 2 SD, mm (range)	6.6 ± 11.4 (0-60)
Localization of Lesion, No. (%) (n = 240)	
Right Upper Lobe	89 (37)
Right Middle Lobe	16 (7)
Right Lower Lobe	42 (17)
Left Upper Lobe	55 (23)
Left Lower Lobe	38 (16)
Radiologic Feature, No. (%) (n = 240)	
Mass Lesion	146 (61)
Solitary Pulmonary Nodule	43 (18)
Consolidation	48 (20)
Cystic	4 (2)
Cavitation, No. (%) (n = 240)	50 (21)
Calcification, No. (%) (n = 240)	17 (7)

The final diagnosis for 199 (82.9%) patients was malignant and the remaining 41 (17.1%) patients were diagnosed benign. The final diagnoses of the patients are given in (Table 2).

**Table 2.** Types and Routes of Final Diagnosis

Diagnosis	TFNA	Mediastinoscopy	Thoracotomy	VATS	Lab.	Clinical Follow-Up	Total
NSCLC	151	6	4	10			171
Unclassified Malignancy			1				1
SCLC	8	2	1	1			12
Metastasis	7		1				8
Lymphoma		2	1				3
Germ Cell Tumor		2					2
Mesenchymal Tumor			1				1
Thymoma		1					1
Tuberculosis	5	2	4		6		17
Hydatid Cyst			2				2
Pneumonia					1	17	18
Abscess						2	2
Hamartoma	1		1				2

Abbreviations: NSCLC, non-small cell lung cancer; SCLC, small cell lung cancer; VATS, video assisted thoracic surgery

One hundred seventy six patients underwent the TFNA procedure only once. Sixty-four patients underwent a second procedure, while 12 underwent a third one (totally 316 procedures in 240 patients). One hundred forty two patients were diagnosed with first trial of TFNA and were not considered for further assessment (diagnosis rate of first TFNA trial was 59%). In the first procedure we established 138 malignant and four benign diagnoses. Ninety eight patients did not have any diagnosis in the first TFNA procedure. Thirty four patients were diagnosed by other routes such as laboratory tests, microbiologic examinations, mediastinoscopy or thoracotomy. Sixty four patients underwent second TFNA in which the diagnosis of 30 patients was determined in the second TFNA (diagnosis rate for second time 47% and total diagnosis rate 72%) (P <0.001). The diagnosis of these additional 30 patients was as follows; 24 inoperable lung cancer, three operable lung cancer, two tuberculosis and one hamartoma. While 22 patients of the 34 remaining patients in the repeated group were diagnosed by other routes such as laboratory tests, microbiologic examinations, mediastinoscopy or thoracotomy, 12 patients were indicated for third TFNA. None of these 12 patients yielded any diagnosis with TFNA and their diagnosis was confirmed by other routes such as laboratory tests, microbiologic examinations, mediastinoscopy or thoracotomy. The other diagnostic tests in this study were carried out according to the clinical status of the patient, radiologic evaluation and physician preference. Twenty-nine (9.2%) pneumothoraces were detected in 316 TFNA procedures. These 29 pneumothoraces occurred in 26 (11%) patients. There were 23 pneumothoraces in the first 240 procedures. Seven out of these 23 pneumothoraces were detected in the 64 patients who proceeded to repeated TFNAs. There were six pneumothoraces (9%) in the second TFNA procedures and it was not different from the pneumothorax rate in the first TFNA trial (P =0.41). However, when we compared the pneumothorax rate versus zero in the second TFNA trial it was significant (P<0,01). In three patients, pneumothorax occurred in the first and second procedures. There was no pneumothorax in the third trial of TFNA. Pneumothorax was detected by immediate CT examination in 25 (86.2%) procedures and by X-rays in four (13.8%) procedures (totally 29 pneumothoraces). Pneumothorax was improved in 20 (69%) by only nasal oxygene supplementation, in four (14%) by simple aspiration and in five (17%) by tube thoracostomy. The existence of pneumothorax was significantly associated with the maximum diameter of the lesion, distance from the pleura, contact to the pleura and the smoking history. On the other hand, pneumothorax development was not associated with age, gender, patient’s position, needle insertion angle, lesion localization and existence of calcification or cavitation (Table 3).

**Table 3.** Factors Influencing Pneumothorax Rate Following Procedures

Factor	Pneumothorax	No Pneumothorax	P Value
Age, mean ± SD, year	63 ± 10	59 ± 12	0.06
Gender, Female/Male	3/26	33/254	0.57
Smoking, Pack/Year	59 ± 32	46 ± 29	0.04
Lesion Size, mean ± 2 SD, mm	40 ± 20	52 ± 22	0.003
Lesion Depth, Mean ± 2 SD, mm	14 ± 12	6 ± 11	0.001
Needle İnsertion Angle, Right/Slopped	26/3	226/61	0.121

## 5. Discussion

TFNA is effective in distinguishing malignant lesions and determining the histopathological type of lung cancer (2, 7, 8). Although CT-guided TFNA is a widely used, safe and practical method in diagnosing local pulmonary lesions, there is no report about the safety and diagnosis rate of repeating the procedure. We have demonstrated that procedure repetition increased the diagnostic yield without an increase in the pneumothorax rate in pulmonary lesions. The diagnostic rate of TFNA for malignant lesions ranges from 76% to 97% (1-3). In our study, the diagnostic rate (72%) was close to the lower limit. One reason was absence of an on-site pathologist to evaluate the adequacy of the specimen. Previous studies have shown that on-site cytological assessment at the time of the procedure reduced the inadequate sampling rate, and increased the diagnostic rate of TFNA (9, 10). Another reason was that we included only fine needle aspiration procedures, which may lead to a decrease in the diagnostic rate. It was reported that the diagnostic rate of core biopsy is higher particularly in benign lesions (1, 11-14). In our study, while we established 165 out of the 199 malignant diagnoses with TFNA, the diagnosis rate for benign lesions was only 18% (7 out of 41 benign diagnosis). We investigated the association between some factors and the diagnostic rate in TFNA. We found only a significant inverse correlation between calcification of the lesion and the diagnosis rate. However, we could not make a conclusion about this result because of the low number of calcified lesions (17/240). We found no association between the lesion size and the diagnostic rate in our study. While some studies reported association between lesion size and diagnostic rate, some found no association (1, 2, 15, 16). We obtained 30 additional diagnoses with the second trial of TFNA procedures in our study. As most of the diagnoses with repeated TFNA were inoperable lung cancer, usually an incurable disease, we prevented these patients from undergoing more invasive diagnostic procedures (such as mediastinoscopy and thoracotomy) by repeated TFNA. We had a diagnostic advantage with repeated TFNA with the cost of an additional six pneumothoraces (9%). Although the pneumothorax rate in the second TFNA was significantly higher than zero, it was not different from the rate of pneumothorax in the first TFNA. However, it is probable that the additional TFNA does not have a diagnostic benefit after the second TFNA. Perhaps the greatest advantage of TFNA is safety. However, pneumothorax is the most common complication associated with the TFNA biopsy. Reported rates range from 7.6% to 46% (2, 4). The pneumothorax rate in the repeated patients of our study was nearly in the lower range of the reported rates (8%). The main reason for this low rate of pneumothorax was the choice to perform a single pass through the lesion. In the previous studies, a strong correlation was reported between pneumothorax and the size and depth of the lesion that are similar to our results (1, 17). In our study, the pneumothorax rate that required chest tube drainage was 17% (5 out of 29 pneumothoraces). The percentage of patients requiring a chest tube after biopsy in reported previous studies varies between 3.3%-43% (2). Up to now, many factors that might affect the pneumothorax rate have been investigated. We observed post procedure pneumothorax more frequently in heavy smokers. This result may be due to the association between the amount of smoking and the risk of emphysema. However, we cannot make such a conclusion, because respiratory function tests and presentations of emphysema were not recorded in the CT imaging of each patient. We found a significant association between pneumothorax rate and the size of the lesion and its distance from the pleura. These findings were parallel with the literature (1, 17). There were some limitations in this study. First, it was retrospective. Second, cytological assessment was not performed at the time of the procedure. This might be the reason of high repetition procedures. In conclusion, this study demonstrated that procedure repetition improved the diagnostic yield without a significant increase in the pneumothorax rate. We consider that TFNA repetition is a safe method with an acceptable complication rate when the first biopsy is nondiagnostic.
